# A reliability assessment of a direct-observation park evaluation tool: the Parks, activity and recreation among kids (PARK) tool

**DOI:** 10.1186/s12889-015-2209-0

**Published:** 2015-09-17

**Authors:** Madeleine E. Bird, Geetanjali D. Datta, Andraea van Hulst, Yan Kestens, Tracie A. Barnett

**Affiliations:** Département de médecine sociale et préventive, École de Santé Publique de l’Université de Montréal, Montreal, QC Canada; Centre de recherche du Centre Hospitalier Universitaire Sainte-Justine, Montreal, QC Canada; Centre de Recherche du Centre Hospitalier de l’Université de Montréal (CRCHUM), Montreal, QC Canada; INRS–Institut Armand-Frappier, Unité d’Épidémiologie et Biostatistiques 531, boulevard des Prairies, Montreal, Laval Québec H7V 1B7 Canada; Department of Epidemiology and Biostatistics, McGill University, Montreal, QC Canada

## Abstract

**Background:**

Parks are increasingly being viewed as a resource that may influence youth obesity and physical activity (PA). Assessing park quality can be challenging as few tools assess park characteristics geared towards youth PA. Additionally, no studies have compared reliability estimates of items assessed in different countries, hindering aims towards generalizable park audit items. Finally, new satellite imaging technology is allowing for desktop identification of parks, however it remains unclear how this compares to direct observation park identification. The purpose of this study is 1) to describe the development and reliability of a youth-oriented direct-observation park audit tool tested in Montreal, Canada, and; 2) to compare reliability estimates of items with those drawn from a tool previously tested in Perth, Australia, with those same items tested in Montreal, Canada.

**Methods:**

Items were drawn and adapted from two existing tools and 13 new items were newly developed for a total of 92 items. Parks were pre-identified using a GIS software and then verified and audited on-site by observers. A total of 576 parks were evaluated. Cohen’s kappa and percent agreement were used to assess the inter- and intra-rater reliability of each item. Inter-rater reliabilities of 17 items drawn from a tool previously tested in Australia were compared.

**Results:**

Eighty-six percent of items had ≥ 75 % agreement and 83 % had kappa coefficients between 0.41 and 1. Among 40 test-retest episodes kappa agreement was relatively high (≥ 0.40) for all but four items. Percent agreement was excellent (≥ 75 % agreement) for all but eight items. Inter-rater reliability estimates of the 17 items tested in Montreal and Perth were of similar magnitude.

**Conclusions:**

The tool is generally reliable and can be used to assess park characteristics that may be associated with youth PA. The items tested in Montreal and Perth are likely generalizable to other urban environments.

**Electronic supplementary material:**

The online version of this article (doi:10.1186/s12889-015-2209-0) contains supplementary material, which is available to authorized users.

## Background

The prevalence of obesity among youth in Canada has been increasing steadily over the past 25 years [1] while Canadian youth lag behind other nations in levels of physical activity [2]. Childhood obesity is a major public health concern; it is associated with chronic health risks during childhood that may last into adulthood [3] as well as adult morbidities such as type-2 diabetes mellitus [4] and cardiovascular disease [5]. Efforts toward curbing obesity among youth, including interventions to increase physical activity, have become a public health priority. Because individual-level interventions for physical activity have had only moderate success [6, 7], attention towards upstream determinants including features of the built environment that may influence physical activity has increased.

Public parks represent a promising area of the built environment for intervention because they have the potential to facilitate or hinder behaviours that are known to affect weight status [8, 9], they are a popular setting for physical activity among youth [10, 11] and they consist of relatively modifiable aspects of the built environment. In addition, in some geographic areas such as the city of Montreal, parks are known to be accessible across a range of socioeconomic status neighbourhoods [12]. Finally, natural experiments have assessed the impact of park improvements on physical activity [13–15]. The park improvements studied include fitness zones appropriate for all fitness levels and individuals aged 13 years or older [13], renovations to soccer fields (e.g., artificial turf to replace dirt fields, and new lighting) [14], and installation of an all-abilities playground as well as new landscaping [15]. These studies demonstrate that public parks are a promising area of the built environment for interventions that promote physical activity at the population level, and point to useful aspects of parks on which to intervene. Indeed, one of these studies [13] demonstrated that park improvements are a cost-effective intervention when considering dollars spent per metabolic equivalent of task gained at the population-level.

The presence of parks has been associated with physical activity among adults and children in the literature, however findings have been mixed as studies are mainly based on perceived accessibility or proximity measures [16]. A number of studies have used qualitative methods to better understand subjective reasons for park utilization among frequent park users [10], and a number of direct observation tools have been developed or adapted from existing tools to objectively assess park characteristics for physical activity [17–24]. Table 1 shows a comparison of the reliability estimates of direct observation park evaluation tools. All but two of the tools were tested in the USA, while two were tested in Australia. All reported that the majority of items were at least moderately reliable or higher (i.e., more than 50 % of items were ≥ 0.40 kappa or ≥ 70 % agreement), which speaks to the general overall reliability of currently available direct-observation park audit tools. Four of the tools [19–21, 24] were developed explicitly to assess park characteristics hypothesized to be associated with physical activity, one of which [19] was developed based on a conceptual model of park characteristics and physical activity [6].

**Table 1:** Comparison of direct-observation park evaluation tools

Tool Name	Author [ref]	Date	Total Items	Number of Test Parks	Test Site	Reliability Estimate	Lowest Estimate	Highest Estimate	% Items ≥ 0.40 kappa or ≥ 70 % agree	Overall Reliability
Recreation Facilities Assessment Tool	Cavnar et al. [17]	2004	61	27	Southeastern USA	Kappa	−0.50	1.00	75 %	0.80 kappa
Public Open Space Tool (POST)	Giles-Corti et al. [24]	2005	49	516	Perth, Australia	Kappa	0.60	1.00	100 %	NA
Physical Activity Resource Assessment instrument (PARA)	Lee et al. [22]	2005	34	22	Kansas City, Kansas and Missouri, USA	10 % Overlap	NA	NA	NA	rs ≥ 0.77
Bedimo-Rung Assessment Tool - Direct Observation (BRAT-DO)	Bedimo-Rung et al. [19]	2006	181	2	New Orleans, Louisiana, USA	Percent agreement	63.60 %	100 %	95.60 %	87.20 % agreement
Environmental Assessment of Public Recreation Spaces (EARPS)	Saelens et al. [18]	2006	646	225	Greater Cincinnati area, Ohio, USA	Kappa, ICC and percent agreement	NA	NA	56 %	65.6 % of 506 items were either kappa/ICC ≥ 0.60 or ≥ 75 % agreement
Path Environment Audit Tool (PEAT)	Troped et al. [23]	2006	40	6	Massachusetts, USA	Kappa (15 of 16 primary amenity items)	−0.03 kappa	1.00 kappa	75 % ≥ 0.04 kappa	≥ 0.49 kappa
						Kappa (7 binary items)				0.19–0.71 kappa
						ICC (3 of 5 ordinal items)	−0.04 ICC	0.84 ICC	43 % ≥ 0.40 ICC	≥ 0.49 ICC
						Percent agreement	34 % agree	100 % agree	85 ≥ 70 % agree	≥ 81 % agreement
Children’s Public Open Space Tool (C-POST)	Crawford et al. [21]	2008	27	19	Melbourne, Australia	Inter- and intra-rater reliability	NA	NA	NA	NA
Community Park Audit Tool (CPAT)	Kaczynski et al. [20]	2012	140	59	Kansas City, Missouri, USA	Kappa	NA	NA	89 % ≥ 0.40 kappa	≥ 0.40 for all but 8 of 56 items where kappa could be calculated
						Percent Agreement	NA	NA	97 ≥ 70 % agree	≥ 70 % agreement for all but 4 items

*NA* Not available, *ICC* Intraclass correlation coefficient

While park audit tools have been useful for identifying aspects of parks that are attractive for physical activity among park visitors [24, 25], only two have focused on park characteristics that may be related to physical activity among youth [20, 21]. Other limitations of the existing park assessment tools include the small number of parks assessed. With the exception of two tools [18, 24], most were tested among a relatively small number of parks (*n* < 60) which may impede reliability as well as external validity of the items tested [17, 19, 23]. As research on parks and physical activity continues, it becomes increasingly important to develop and assess the reliability and validity of park measurement items in a variety of contexts to facilitate the comparability of research results [26] and help minimize measurement error. To date, no items on any of the park evaluation tools have been assessed in more than one geographic context. Finally, as global satellite imaging, such as Google Earth or geographic information systems (GIS) in general, improves, desktop assessment of parks holds promise for research because it may be a cost effective way to identify and evaluate parks [27]. However, it remains unclear whether the images are accurately identifying existing parks.

Reliable evaluation of park features and characteristics that may be appealing to youth is a foundational step in efforts toward identifying those park characteristics that may be associated with physical activity among youth. There is a need for more reliability assessments of direct observation audits of park characteristics. Indeed, a recent literature review on the built environment, physical activity and obesity called for more specific assessment of parks through direct, objective and systematic observation of details such as the quality of the amenities, or the “micro-scale” variables, in parks [28].

For the present study, the original intent was to use an existing park direct-observation tool (the Public Open Space Tool [29], or POST), however it became clear that no single existing tool available at the time of the study (2007) was able to meet the needs of the overall study objectives in terms of efficiency and relevance. At the time of the present study, the two park audit tools [20, 21] that were developed for assessing parks for physical activity among youth were not yet available. The park audits in the present study were embedded in larger detailed neighborhood direct-observation audits of a 500 m walking network buffer around the homes of study participants, therefore requiring thorough yet efficient park evaluations given that the evaluation of the walking network buffer and the three closest parks near the homes of the youth involved in the study had to be conducted during a one-day visit. The relevance of items was also important regarding the efficiency of the audit, as a number of items on existing tools at the time were not relevant for the study context. Thus, a new study tool, drawing on items and methods from different existing tools, and incorporating new items and response schemes, was developed for the present study. Given these changes, an independent reliability study was warranted; furthermore, the tool described herein may be useful for replication and application in other studies.

The objectives of the current study are therefore twofold: 1) to describe the development and reliability of a youth-oriented direct-observation park audit tool tested in Montreal, Canada, and; 2) to compare reliability estimates of items with those drawn from a tool previously tested in Perth, Australia [24, 29], with those same items tested in Montreal, Canada. A secondary finding that emerged in the process regarding the reliability of park identification via GIS will also be briefly discussed.

## Methods

### Study context

The park evaluation tool was developed for the QUALITY Neighbourhood Study, an adjunct to the QUALITY (Quebec Adipose and Lifestyle Investigation in Youth) Cohort study, an ongoing longitudinal investigation of the natural history of obesity and cardiovascular risk among youth with a parental history of obesity. A detailed description of the study design and methods is available elsewhere [30]. Written informed consent was obtained from the parents, and assent was provided by the children. The Ethics Review Boards of CHU Sainte-Justine and Laval University approved the study. Parks were evaluated for QUALITY participants residing in the Montreal Census Metropolitan Area (*n* = 512), up to three parks (*n* = 576 parks) in a 1000 m walking buffer zone around participants’ exact addresses were audited. The park audits were embedded in a larger neighbourhood direct-observation evaluation around the homes of QUALITY participants.

### Tool development

The study team utilized existing direct observation tools as the basis on which to create a new tool adapted to the Canadian context. The team assessed all available park audit tools published until 2007 [17–19, 29], the year the study began. Each item contained in the extant tools was assessed for (i) its reported reliability and (ii) its potential to measure park characteristics that are likely to appeal to youth (i.e., between 5 and 18 years) physical activity (e.g., installations for team sports, swimming pools) based on group discussion by the research team. Of the tools assessed, the Bedimo-Rung Assessment Tool-Direct Observation (BRAT-DO) [19] and the Public Open Space Tool (POST) [24] contained items that had demonstrated reliability and were relevant for inclusion for a youth oriented park evaluation tool. The result was a 92-item youth-oriented park audit tool, the Parks, Activity, and Recreation among Kids or PARK Tool (see Additional file 1 to view the tool). The PARK Tool was developed to assess 5 conceptual domains based on the Bedimo-Rung conceptual model of parks and physical activity [6] that may be important for youth: 1) Activities (17 items and 39 sub-items); 2) Environmental Quality (9 items and 3 sub-items); 3) Services (10 items and 2 sub-items); 4) Safety (6 items), and; 5) General Impression (6 items).

The present tool drew the majority of its items from the POST and the BRAT-DO including 24 items that appear in both tools, 12 items that appear exclusively in the POST and 43 items that appear exclusively in the BRAT-DO. In addition, thirteen items were newly developed, for a total of 92 items. Almost all of the items were moderately to significantly re-worded for the PARK tool and response scales were changed. For example, the POST has an item, “Are picnic tables present?” with response scale 1 = yes; 2 = no, whereas in the PARK tool, the item is worded as, “Picnic tables”, with response scale 1 = yes, in usable condition; 2 = yes, but unusable; 3 = no. More details on the changes made to items are described below. Most of the qualitative items regarding the activity installations (e.g., accessibility, condition and restriction) were drawn from the BRAT-DO; the qualitative items regarding the accessibility, condition and restriction of water-sprinklers, skate parks and schoolyards were newly added. All thirteen new items were included to assess features of parks that would likely appeal to or be relevant specifically to youth physical activity in parks. These items include the presence of schoolyards, skate parks, water sprinklers, and qualitative general impression items such as overall safety and appeal for youth.

The response options on the tool include binary yes/no, or present/absent responses (*n* = 61), 3-point scale items (1 being the most favourable response and 3 being the least favourable response, e.g., for presence of graffiti: 1 = none, 2 = some, and 3 = a lot; except for the pool length item which had three options for pool length) (*n* = 24), 4-point scale items, such as the cleanliness of water sprinklers where 1 = very clean, 2 = clean enough, 3 = not at all clean, and 4 = impossible to evaluate (*n* = 2), 5-point scale items, such as water sprinklers condition where 1 = no deterioration, 2 = presence of deterioration without need for repairs, 3 = significant deterioration requiring repairs, 4 = under construction and 5 = impossible to evaluate (*n* = 3), and two text responses.

During pilot testing, items drawn from the POST and BRAT-DO were changed and adapted to the PARK tool. Changes were made to items that had systematically poor agreement, that were less relevant to the study site, that were confusing to observers, or that required more detail. For example, in the PARK tool, unlike in the BRAT-DO and POST, every activity installation item had three adjoining qualifying items added to them: check if the installation is accessible, in good condition, and restricted. The POST and BRAT-DO both contained a number of items regarding water features, including beachfront features, which were not applicable for the study site. These items were consolidated into one primary and one sub-item: Important body of water present, and if yes, are there sportive aquatic activities present. The POST and BRAT-DO also had a number of items about dog related amenities (e.g., “Are dog litter bags provided?” on the POST and “Are there any signs specifying that dog owners must dispose of pet droppings?” on the BRAT-DO) that were not included except for a modified item that asks the observer to check yes if dogs are not allowed in the park. Safety items from the POST, such as, “From the centre of the POS [public open space], how visible are surrounding roads” were modified to “At least 1 street visible from the centre of the park”. All item modification and development were conducted via an iterative process that was conducted at the beginning of the tool pilot stage (e.g., after a pilot run some items were modified if they caused confusion for observers) and the final items were agreed upon through discussion and consensus by the study team. The tool was piloted among observers from diverse ethnic backgrounds in their early twenties (*n* = 12).

### Park identification and sampling

Park identification was conducted using a two-stage process. First, a geographic information system (GIS) was used with land use information from CanMap (Digital Mapping Technologies, Inc., 2007) by a trained geographer where a ‘parks and open space’ category was used to identify the three closest parks within a 500 m walking network buffer centered on the exact addresses of the youth participants in the QUALITY Cohort. If no parks were found within the 500 m buffer zone, the closest park present within a 1000 m walking network buffer zone was identified. A park was defined as a public open space large enough to play a game of catch or roughly half the size of a soccer field (e.g., approximately 50 m long by 30 m wide). This included parks adjacent to schools and schoolyards. If the observers were not sure, they were instructed to look for the name of the park on a sign. Spaces that were exclusively passive, e.g., not large enough or equipped for physical activity (e.g., a concrete area with park benches only) were excluded, as were parks that were exclusively equipped with amenities and installations for children 5 years and under, and where there was a sign explicitly stating that the area was restricted for children 5 years and younger. Cemeteries and golf courses were also excluded.

Each park identified using the GIS was assigned a unique identification number and indicated on maps provided to observers on the observation day. On observation days, parks were also identified on-site using a ‘seek and assess’ procedure where observers systematically walked all the street segments in the 500 m buffer zone to identify parks that were not reported in CanMap. When a non-reported park was identified by observers they drew its spatial boundaries on the map provided and highlighted the nearest intersection. The percentage of parks correctly identified using CanMap was calculated in order to compare the identification of the existence of parks through direct observation and desktop park identification. A total of 576 unique parks were assessed. Sixty-four percent of the sample, or 345 of 576 parks, were pre-identified using CanMap, while 36 % were identified on-site. Park assessment data were directly imported into a database from personal digital agendas (Pocket PC iPaq 110), thereby eliminating data entry errors. The parks were audited during clement weather between the hours of 8:00 and 17:00 in 2008 (76 %), 2009 (21 %), and 2010 (3 %), between the months of June and December. No parks were evaluated when there was snow coverage on the ground.

### Observer training

Nine observers were recruited for data collection. Observers were between the ages of twenty-one and thirty, seven female and two male, recruited through University employment services. Observers were mostly undergraduate or recent graduate Kinesiology or Community Design students. Observer training occurred over 9 non-consecutive full-days (9:00–17:00) beginning in May 2008. The 9-day training on park audits was embedded in the larger neighbourhood environmental audit around the homes of the QUALITY Study participants. On the first day of training, observers were introduced to the purpose of the study and attended a presentation of the observation tool that contained photo illustrations of answers for each question. Observers were provided with the observation tool manual and requested to read it thoroughly prior to on-site evaluation. On the five subsequent training days, observers and trainers began running independent on-site test observations in various non-study parks in the Montreal area. Following each on-site training session, observers met with the trainers in the park and later at the research centre to compare answers. In cases of discordant answers, the group would return to the area of the park in question to identify what the “correct” answer should be based on the trainer’s response, considered the gold standard. Following each on-site training day, items on the PARK tool were revised and adjusted in efforts to improve clarity and inter-observer reliability. The most common change was a reduction in the number of response options.

During the iterative on-site observer training sessions, a pen-and-paper version of the tool was used to record answers. On day 7 of training, the observers began to use the personal digital agendas containing a programmed Microsoft Excel spreadsheet with a cell drop-down function to record answers to the revised tool. Once again discordant answers were discussed following park audits. This process was repeated with the digital agendas for days 8 and 9. On day 10, observers began evaluating parks around the homes of the QUALITY participants.

### Observer reliability and validity assessment

Each park was audited by two observers who evaluated the parks independently but at the same time on the same day of the week. Observer pairs were assigned on the morning of every day of observation, so that the pairs were not always the same two individuals. Inter-rater reliability was estimated by comparing the responses from observer pairs for each park. Intra-rater reliability was assessed using test-retest methods wherein the same observer audited a park on two separate occasions, and the results of the independent audits of the same park by the same observer on different occasions were compared. The median number of days between observers’ first and second park audit was 61, with a minimum of 3 and a maximum of 448 days, and a mean of 163 days. All observers re-evaluated approximately 4 parks each on a separate occasion. A total of forty parks were evaluated twice by the same observer, or 7 % of the total park sample. Reliability estimates of a test-retest can only be calculated using complete data with response item variation. For example, if there is no tennis court in any of the parks that were re-audited by the same observer, then the intra-rater reliability of this and associated items (e.g., tennis court condition) cannot be evaluated. The data collected for training purposes was not used. If a park audited for training purposes was also in the study sample, the park was re-evaluated for the study.

### Comparison of reliability estimates between POST and PARK tools

Reliability of the POST was assessed in 2003 among 516 parks in Perth, Australia. Results are published and available on an institutional website [31]. Thirty-six items on the PARK Tool were drawn from the POST; however only seventeen items could be directly compared with items assessed in Montreal, Canada, because a number of items drawn from the POST had been substantially modified for the PARK Tool, and because not all items on the POST used the kappa coefficient to estimate reliability (intraclass correlation coefficient was used instead). Inter-rater reliability of the POST was estimated by calculating Cohen’s kappa and percent agreement between raters. An item-by-item comparison between items shared with the BRAT-DO could not be conducted because no corresponding item-level reliability estimates were published for the BRAT-DO. Comparison of reliability estimates were done visually and qualitatively using established cut-offs [18, 32].

### Statistical analyses

Frequency distributions were examined for categorical variables. Inter-rater reliability was estimated using percent agreement per measurement episode as well as Cohen’s kappa to account for chance agreement. Cut-offs for percent agreement categorization were implemented according to criteria established by Saelens and colleagues [18] as “good to excellent” (≥ 75 %), “moderate” (60–74 %), or “poor” (< 60 %). Although the meaning ascribed to the value of a kappa statistic may change according to subject area, Landis and Koch [32] provide the following guidelines which are used here: > 0.80–1 = almost perfect agreement; > 0.60–0.80 = substantial agreement; > 0.40–0.60 = moderate agreement; > 0.20–0.40 = fair agreement; 0–0.20 = slight agreement, and; < 0 = poor agreement. Simple unweighted kappas were calculated for all dichotomous variables and weighted kappas were calculated for all categorical variables where possible (kappa cannot be calculated when there is no response variation, i.e., where all observers agreed on the response). Intra-rater reliability was estimated using a test-retest method. Again, Cohen’s kappa and percent agreement were calculated between the first and second assessment of the same park by the same observer on a different measurement occasion. All analyses were performed using SAS, version 9.2 (Cary, North Carolina).

## Results

### Inter-rater reliability and validity assessment

Eighty-six percent of items across all 576 parks demonstrated ≥ 75 % agreement, indicating good to excellent overall agreement. A small number of the activity installation qualifying items (tennis restriction, basketball condition, track condition, and pool length) had < 75 % agreement, with the lowest percent agreement being 70.2 % for pool length. Other items with low agreement include presence of shade, graffiti and litter (67.5, 69.3 and 67.3 % agreement respectively). The item presence of pedestrian safety features had low percent agreement (73.8 %) and the subjective general impression items, all had low agreement (ranging from 58.2 to 61.1 %) except for attractive for bicycling (81.5 %). See Table 2 for a summary table of percent agreement by domain. Among the items for which kappa coefficients could be calculated (*n* = 79), 85 % were found to be between > 0.40 and 1 (28 % moderate agreement, 27 % substantial agreement, and 30 % almost perfect agreement). Items for which there was low agreement include the activity installation condition items for tennis condition (kappa = 0.18), basketball condition (kappa = 0.25), trail condition (kappa = 0.26), 6-plus play area condition (kappa = 0.30), multi-use area condition (kappa = 0.24) and school yard condition (kappa = 0.17). The skate park restriction item also had poor agreement (kappa = 0.10), as did the presence of vandalism item (kappa = 0.22). Two of the general impression items had poor agreement: overall safe (kappa = 0.35) and overall attractive/pretty (kappa = 0.36). Kappa coefficients could not be calculated for 11 of the items due to a lack of response variation (e.g., for the item “presence of aquatic activities in a pond”). See Table 3 for a summary table of Cohen’s kappa estimates by domain. Additional file 2 reports the inter-rater reliability results of all items on the tool.

**Table 2:** Inter-rater reliability (percent agreement) by domain

Domain	# Items	Average Agree	Highest Agree	Lowest Agree	# Items ≥ 70 % Agree	% Items ≥ 70 % Agree
Activities	55	92 %	100 %	70 %	55	100 %
Environmental Quality	11	83 %	100 %	67 %	8	73 %
Services	12	92 %	98 %	81 %	12	100 %
Safety	6	80 %	88 %	63 %	5	83 %
General Impression	6	65 %	82 %	58 %	1	17 %
Overall	90	82 %	94 %	68 %	81	75 %

Note: 2 items are text items not included in this table

**Table 3:** Inter-rater reliability (Cohen’s kappa) by domain

Domain	# Items	Average Kappa	Highest Kappa	Lowest Kappa	# Items ≥ 0.40 Kappa	% Items ≥ 0.40 Kappa
Activities	55	0.65	1.00	0.10	38	84 %
Environmental Quality	11	0.63	1.00	0.22	10	91 %
Services	12	0.80	0.92	0.67	11	100 %
Safety	6	0.58	0.65	0.45	6	100 %
General Impression	6	0.47	0.59	0.35	4	67 %
Overall	90	0.63	0.83	0.36	69	88 %

Note 1: 2 items are text items not included in this table

Note 2: 10 items in the Activities domain and 1 item in the Services domain did not have enough response variation to calculate Cohen’s kappa

### Intra-rater reliability

There were a total of 40 test-retest episodes among all 9 observers (that is, 40 parks were audited twice by the same observer on two different dates). As mentioned above, because of the relatively small number of parks audited twice by the same observer on a separate occasion, only 44 items had complete data for audit one and two of the same park. Overall, kappa agreement between test time one and two were relatively high (> 0.40) for all but four of the items for which kappa could be calculated (see Table 4). Percent agreement was excellent (≥ 75 % agreement) for all but eight items (see Table 5). There were seven items that had both poor intra-rater reliability and poor inter-rater reliability based on % agreement. The presence of graffiti had poor intra- and inter-rater percent agreement (67.5 and 69.3 %, respectively), as did the presence of litter (52.5 and 67.3 %, respectively). Traffic calming measures had poor percent agreement for both intra- and inter-rater agreement (55 and 63.2 %, respectively), as did pedestrian safety features (65 and 73.8 %, respectively). Three general impression items, overall appealing for youth, overall safe, and attractive for walking had poor % agreement for both intra-and inter-rater agreement (60 and 60.1 %, respectively for overall appealing for youth; 70 and 60 %, respectively for overall safe, and; 67.5 and 66.6 %, respectively for attractive for walking). Additional file 3 reports the intra-rater reliability results of all 44 items on the tool.

**Table 4:** Intra-rater reliability (Cohen’s kappa) by domain

Domain	# Items	Average Kappa	Highest Kappa	Lowest Kappa	# Items ≥ 0.40 Kappa	% Items ≥ 0.40 Kappa
Activities	15	0.74	0.94	−0.03	13	93 %
Environmental Quality	9	0.60	1.00	0.38	8	89 %
Services	8	0.66	0.73	0.63	7	100 %
Safety	6	0.49	0.74	0.32	4	67 %
General Impression	6	0.62	0.84	0.48	6	100 %
Overall	44	0.62	0.85	0.36	38	90 %

Note: kappa could not be calculated for 1 item in the Activities domain and 1 item in the Services domain due to a lack of response variation between observers

**Table 5:** Intra-rater reliability (percent agreement) by domain

Domain	# Items	Average Agree	Highest Agree	Lowest Agree	# Items ≥ 70 % Agree	% Items ≥ 70 % Agree
Activities	15	94 %	98 %	83 %	15	100 %
Environmental Quality	9	80 %	100 %	53 %	7	78 %
Services	8	83 %	88 %	73 %	8	100 %
Safety	6	75 %	88 %	55 %	4	67 %
General Impression	6	74 %	93 %	60 %	4	67 %
Overall	44	81 %	93 %	65 %	38	82 %

### Comparison of kappa results from items on the PARK tool and the POST

Inter-rater reliability estimates for 17 items were compared and found to be of a similar magnitude (see Table 6). Fourteen of the seventeen items compared were ≥ 75 % agreement on both the POST and the PARK tool. Percent agreement was not available for one item on the POST (Picnic tables present). For two items (presence of graffiti and litter), the PARK tool had moderate agreement (69.34 and 67.30 % agreement, respectively) while the POST had good to excellent agreement (78.26 and 76.00 % agreement, respectively) for these same items. The kappa coefficients fell in the same ranges for 10 of the seventeen items (4 items had kappa > 0.80, 3 items had kappa > 0.60–0.80, and 3 items had kappa > 0.40–0.60 for both items on both tools). Seven items were not in the exact same range, although all were similar in range (e.g., drinking fountains present had almost perfect agreement on the PARK tool and had substantial agreement on the POST, or sufficient lighting had moderate agreement on the PARK tool and substantial agreement on the POST) and none differed by more than one qualifying category. In other words, there were no items shared between the POST and the PARK tool that had vastly different reliability estimates.

**Table 6:** Comparison of inter-rater reliability of items shared between the PARK tool and the POST

	PARK ^b^ (*n* = 576)		POST ^a^ (*n* = 47)	
Item	Kappa	% Agreement	Kappa	% Agreement
6+ Play Area Present	.935	97.74	1.00	100.00
Large Body of Water Present	.918	98.95	.876	97.70
Drinking Fountain Present	.918	94.77	.746	87.20
Public Toilets Present	.822	92.15	.849	95.60
Picnic Tables Present	.855	92.68	.956	--
Parking Present	.728	86.19	.744	87.20
Garbage Bins Present	.811	97.91	.691	93.60
No Dogs Allowed Sign Present	.767	88.33	.849	95.70
Public Transportation Present	.759	88.48	.539	76.00
Sitting Benches Present	.679	94.93	.877	97.70
Chalet/ Change Room Present	.673	91.46	1.00	100.00
At Least 1 Street Visible from Center	.644	88.49	.789	97.90
Trail/ Walking Path Present	.602	80.80	.707	85.10
Sufficient Lighting for Park	.591	83.28	.675	85.10
At Least 1 House Visible from Center	.554	86.74	.486	89.30
Graffiti Present	.514	69.34	.565	78.26
Litter Present	.417	67.3	.495	76.00

^a^ Data printed with permission from the author, B. Giles-Corti

^b^ All categorical items have been dichotomized

## Discussion

A reliable direct observation park evaluation tool that may be used to assess associations between park characteristics and youth physical activity was successfully developed. The items on the PARK Tool are reliable between observers and over time. In addition, the items drawn from the POST demonstrated very similar reliability estimates despite differences in location (Montreal, Canada vs. Perth, Australia), time of observation and observers. Because of the very similar and acceptable agreement of the items shared between the POST and the PARK tool, it can be argued that these items are likely generalizable across at least two very different geographic contexts and may be reliable in other urban contexts as well. The only item shared between the two tools that had slightly lower reliability (moderate agreement) was ‘presence of litter’. One implication of this may be that this item may never achieve very high agreement and that a kappa of > 0.40 can be considered quite good for this particular subject matter. Although for only a small subset of the overall items, this study provides the first known comparison between reliability estimates of the same items on tools tested in different countries. Drawing items from existing tools and comparison of reliability results with the original tool has been encouraged [33] as it will facilitate comparison between studies from different regions, helping to draw robust conclusions about park characteristics and health behaviours or outcomes.

As expected, some subjective items (e.g., overall safe, overall attractive/pretty, and vandalism present), demonstrated generally lower reliability estimates than objective items for inter-rater reliability. The overall safe and overall attractive/pretty items may need to be modified because of the large variation in subjective perceptions of attractiveness and safety between raters. Modification to the overall safe item could include a link to the objective safety items already checked in the tool. Thus, to rate the park as ‘very safe’, the observer would have had to have checked ‘yes’ to a minimum of two of the following safety items: sufficient lighting; at least 1 street visible from the center of the park; at least 1 house visible from the center of the park. To reply that the park is ‘safe enough’, the observer would have had to have checked ‘yes’ to one of the above listed safety items. If the park is rated as ‘not safe’, then none of the above safety items would have been present. For the overall attractive/pretty item, an objective benchmark could be that there is a decorative piece present that is not in disrepair (such as a sculpture/statue or a fountain) or the presence of a garden. Vandalism of park features may be difficult to identify because raters may not be able to recognize the difference between general wear and tear or poor upkeep of park installations and explicit vandalism. This item may also need to be modified. One way this item may be modified is to add objective qualifiers such as presence of broken or damaged installations. The condition of park features is likely an important determinant of park visitation by youth, and signs of civil disobedience in parks are variables worth attempting to capture. How this is done, however, may require more objective measures such as through the litter and graffiti items, or items for a specific type of litter such as the presence of empty alcoholic beverages (bottles or cans). Other objective items that capture civil disobedience should be assessed for their reliability in park audit tools, for example presence of broken glass or syringes.

Collectively, results from this and other direct-observation park evaluation tool reliability studies [18, 23, 24] demonstrate that subjective items tend to generate lower reliability estimates from independent observer pairs. Methods for reliably assessing subjective aspects of park features need to be explored further. This may include changes to subjective items’ definitions to include more objective benchmarks in order to help guide responses, such as those suggested above. Enhanced observer training, such as those outlined by Zenk, Schultz, Mentz et al. [34] may also be a way to improve reliability estimates of subjective items, such as requiring observers to successfully pass an observation reliability test on test sites prior to data collection. For example, if observers are unable to achieve an overall reliability of a 0.60 kappa or 70 % agreement after training, then they are not retained for the study. Finally, a collection of items that attempt to measure different facets of the same construct may improve reliability estimates of subjective items to allow for more thorough analyses, such as through the use of factor analysis to identify which items adequately measure subjective concepts.

A small number of items demonstrated poor percent agreement on both the intra- and inter-rater assessments including the presence of graffiti and litter, traffic calming measures and pedestrian safety features, and overall appealing for youth, overall safe and overall attractive for walking. These items may have had poor agreement for the intra-rater assessment because of substantive changes to the environment, e.g., litter and graffiti could have been removed and traffic calming and pedestrian safety features could have been installed. Whereas for the poor inter-rater agreement for these items, it may be difficult for raters to adequately assess or identify graffiti, litter and pedestrian safety and traffic calming features. Regarding the poor intra- and inter-rater reliability of the general impression items, substantive changes in the parks could again explain the poor intra-rater agreement between visits while the very subjective nature of these items may make achieving a high inter-rater agreement difficult.

A secondary finding regarding the large number of parks that were not identified by GIS warrants a brief discussion. The exercise of pre-identifying parks using geocoded satellite/administrative data prior to observers entering the field facilitated the on-site evaluation process. However, only 64 %, of the sample was pre-identified, meaning that observers, using on-site methods, newly identified over one third of the parks evaluated. In addition, some of the parks that were pre-identified were found to have different boundaries when identified on-site. When this was the case, observers would modify the park on the observer map to reflect its actual size or shape. There were no pre-identified parks that were then not found, however a very small number of areas that were pre-identified using GIS were later identified on-site as golf courses or cemeteries. There were no systematic differences between the types of parks identified via GIS or on-site, other than size. There were four very large parks in the sample (> 200 000 m^2^) that were all identified by GIS. The on-site identification of parks allowed for a more valid sample of the parks of interest in terms of number, location and size, suggesting that studies using satellite images for park identification should validate their findings using on-site verification with a representative sample of the parks. Seeing as direct observation requires significantly more resources than desktop park evaluation [27, 35], further research should be conducted to work toward improving park identification through satellite images and thus improve capabilities to conduct reliable desktop assessments.

### Study limitations

There were a number of study limitations. First, the study was not initially designed to assess test-retest reliability, resulting in a low number of test-retest occurrences and an inability to assess intra-rater reliability for all items. The wide range of days between tests was not controlled for and this may have compromised the validity of the test-retest results. The median number of days between tests was 61 days and the mean number was 163, or approximately 5 months. This time lag likely resulted in non-differential misclassification thus underestimating the test-retest reliability, along with an increasing chance of substantive changes to park features between the first and second tests, however likely only for the more transient items such as graffiti, litter and vandalism. Nevertheless, the overall high kappa and percent agreement between test time one and two attest to the general validity of the time interval between the test-retest conducted here.

Although experts in the research field assessed the PARK tool, it was not assessed by community stakeholders or park professionals, nor were tweens consulted. The tool therefore may not capture all aspects of parks that are interesting for physical activity among tweens, and may not capture items that are important for community members. In addition, at the time of this study’s tool development (2007), the other two park audit tools developed for youth activity [20, 21] were not yet available. Any future direct audits of parks for physical activity among youth could consider the items on these other two tools when either developing a new tool or choosing to use an existing one. Further, comparison of items across geographic contexts could only be conducted on a small subset (17/92) of the items on the PARK tool, limiting the generalizability of the PARK tool to other contexts for these items only. Nevertheless, it is an important first step in assessing the reliability of direct observation park audit items in different geographic contexts.

Finally, although the PARK tool aims to assess features of parks that are conceptually attractive for youth, this has not as of yet been validated. Future analyses are planned to assess construct validity in order to ascertain whether the items of the PARK tool apply to youth physical activity. Future research should validate the tool for youth physical activity by exploring the relationships between park characteristics and their associations with physical activity, body mass index, and other health outcomes, among a youth population.

## Conclusion

Youth are an important target population for increased physical activity due to concerns of overweight and obesity and a lack of physical activity among this population. The results establish the overall reliability of the PARK tool when appropriate training, such as that described above, has been provided to observers. The tool can be recommended for use to assess park characteristics that are considered appealing for youth physical activity according to the Bedimo-Rung framework, however the tool’s validity remains to be established. Future research should estimate the reliability of the items shared between the POST and PARK tool in different geographic regions and compare them with the results found here. In addition, future research should estimate the reliability of the items on the PARK tool in other geographic regions.

## Additional files

Additional file 1:
**The Parks, Activity and Recreation among Kids (PARK) Tool.** This contains the complete and final direct observation tool developed and tested for reliability in Montreal, Canada. (XLSX 14 kb) Additional file 2:
**Kappa and percent agreement results for all items on the PARK Tool.** This contains the kappa and percent agreement estimates for all the items on the PARK Tool. (XLSX 13 kb) Additional file 3:
**Test-retest results for intra-observer reliability.** This contains the intra-observer reliability estimates for 41 items on the PARK Tool. (XLSX 11 kb)
